# Analysis of Charge Parameter Characteristics of Graphene Partial Discharge Sensor Based on First-Principles Study

**DOI:** 10.3390/mi17050530

**Published:** 2026-04-27

**Authors:** Huiyuan Zhang, Pengfei Jia, Ming Nie, Jiayun Zhu, Zhiyuan Li

**Affiliations:** 1 China Electric Power Research Institute, State Grid Corporation of China, Beijing 100192, China; 2 State Grid Economic and Technological Research Institute, State Grid Corporation of China, Beijing 102209, China

**Keywords:** graphene, partial discharge sensors, electrical charge properties, first-principles, electron transport

## Abstract

With the proposal of transparent power grids, advanced sensor research has become a hot topic. A partial discharge (PD) sensor is a specialized device that captures electrical signals generated by partial discharge phenomena in power system insulation, enabling real-time monitoring of insulation status and early warning of potential faults. However, the detection sensitivity and signal transmission efficiency of conventional PD sensors are constrained by the intrinsic properties of their sensing materials. This paper focuses on the improvement of the PD sensor using advanced graphene sensing materials. First-principles calculations were performed to evaluate the key charge parameters of the PD sensor. The microstructure model of the PD sensor is constructed, and the charge parameter properties of the graphene partial discharge sensor are calculated and revealed under simulated electric field. Then, the charge transport characteristics of the PD sensor are simulated. The results reveal that the graphene-based sensor exhibits a significantly enhanced transport coefficient—approximately 66% higher than that of conventional sensor materials. Subsequent experiments revealed the better signal transmission of the graphene PD sensor, which outperformed the traditional sensor by 40%. This study provides a microscopic theoretical reference for optimizing electrode plate materials from the atomic level and the device level, which is of great significance for the design and development of high-performance PD sensor power grids.

## 1. Introduction

For the improvement of new power systems, such as transparent power grids and digital power grids, advanced sensors can be used as nerve endings to make power systems more transparent. In power systems, high-voltage electrical switchgear provides a great guarantee of operation and stability [1]. However, the switchgear operation process is highly susceptible to different degrees of partial discharge problems, which lead to insulation faults in the power system [2]. Partial discharge monitoring is essential for power switchgear, in which PD sensors play a vital role [3].

Conventional methods for partial discharge sensing are broadly categorized as either electrical or non-electrical detection techniques [4], including the pulse current method, ultra-high frequency (UHF) method, transient earth voltage (TEV) method, and ultrasonic method [5]. As an important testing equipment, the improvement of PD sensors has been the focus of research for a long time. Uwiringiyimana et al. [6] designed an ultra-wideband antenna-based PD sensor that exhibits excellent performance at suppressing interference at low frequencies. Saber et al. [7] developed an Ag/Au bimetallic grating-based approach for optical partial discharge detection, demonstrating enhanced sensor sensitivity. Ren et al. [8] developed a miniature built-in discharge sensor using a multi-channel silicon photomultiplier (SiPM) array. Liu et al. [9] proposed and designed a turret-electrode PD sensor. The transformer’s turret functions as a waveguide, while a grounded loop electrode serves as the coupling component. These improvement methods are limited by the sensing structure and conventional sensing materials, which lead to poor coupling performance of the PD signal. This study focuses on the improvement of sensing materials and aims to investigate PD sensors utilizing advanced sensing material.

Graphene, with its high electron mobility, is one of the best conductive materials at room temperature and has already been widely used as sensing material in sensors [10]. Raad et al. [11] introduced a graphene-based core–shell spherical particle configuration for the development of a dual-band refractive index sensor. Zhao et al. [12] presented a new micro-pressure sensing array based on graphene, featuring a dual-layer mesh architecture. Jiang et al. [13] presented a phased-array antenna sensor utilizing a highly conductive graphene-assembled film for enhanced performance. Xu et al. [14] fabricated a flexible resistive pressure sensor featuring a mesh-convex platform microstructure with graphene as the conductive layer, effectively overcoming the limitations of conventional rigid sensors, such as excessive bulk and limited flexibility. However, the application of graphene-based materials has not yet been explored in the development of PD sensors. Therefore, this study focuses on enhancing the electrode plate material for PD sensors by incorporating graphene with conventional copper substrates. Building upon traditional copper electrode designs, this integration aims to improve the overall performance and detection capability of the sensors.

With the rapid development of computational power, computer simulations offer a powerful means to not only investigate material properties but also to uncover the microscopic mechanisms behind them, findings that are instrumental in guiding experimental studies. First-principles studies, which provide a powerful tool to study the electrical charge properties of materials, are currently widely used in materials research. Aasi et al. [15] investigated the sensing performance of five typical SF_6_ decompositions (including H_2_S, HF, SO_2_, SO_2_F_2_ and SOF_2_) on the novel monolayer PdPTe using first-principles calculations. The findings of this study enable innovative methods for monitoring and assessing the insulation status in SF_6_ switchgear. Al-Muhimeed et al. [16] explored the electronic, mechanical and optical properties of Cs_2_SeX_6_ (X = Cl, Br, I) by first-principles analysis, focusing on this novel double perovskite material for solar cells and photovoltaic applications. Lin et al. [17] investigated the effect of chemical composition on the properties of a high-entropy Co_X_Ni_50__-__X_Fe_25_Cr_25_ alloy using first-principles calculations. First-principles studies can predict and explore the structural and physical properties of graphene PD sensor.

Recent first-principles studies have begun to explore the use of graphene-based materials for PD sensor applications. Zhang et al. [2] conducted an analysis of PD sensor electrode plates using DFT calculations, demonstrating that double-layer graphene copper-clad electrodes exhibit superior electrical transport capacity. However, their study focused primarily on material selection. Density functional theory (DFT) studies have explored the application of novel materials, such as Pd/Rh-doped h-BN [18] and Sc/Ti-decorated h-BN [19], for gas sensing in transformer fault detection. However, their primary focus has been on chemical adsorption mechanisms for gas molecule detection, leaving the charge transport dynamics critical for PD sensing largely unexplored. In contrast, the present study establishes a direct link between atomic-level graphene charge parameters and macroscopic PD sensing performance, providing a comprehensive multi-scale theoretical framework from atoms to device.

Therefore, considering that the application of PD sensors involves the coupled transport at the charge level, it is necessary to further analyze the charge parameter characteristics of the graphene PD sensor, and verify that graphene can improve the sensitivity and detection ability of the PD sensor from the charge perspective. The novel contributions of this paper are as follows:

(1) Based on a first-principles study, the charge parameter analysis method is proposed to analyze the new graphene PD sensors under an electric field.
(2) An analytical method for the electrical transport properties of PD sensors is presented under an electric field from the charge angle.
(3) The microscopic theoretical calculations are combined with a macroscopic experiment, which further verifies the feasibility of the microscopic analysis and can provide a theoretical basis for the design of graphene PD sensor.

## 2. First-Principles Study

The first-principles study is based on the Coulomb interaction theory of electrons and nuclei to calculate the structure, charge density and energy of a molecule or atom. This approach allowed for the derivation of various physical properties of the graphene PD sensor material, most notably through modeling via the Schrödinger equation [20], which is shown in Equation (1):

(1) Hψ(r,R)=Eψ(r,R)

where *Ψ* corresponds to the wave function, **r** represents coordinates of electrons, *E* represents the energy, **R** represents coordinates of atomic nucleus, *H* represents the Hamiltonian quantity which can be expressed as Equation (2):

(2) $$H=-\sum_{I}\frac{h^{2}}{2M_{I}}\nabla_{I}^{2}-\sum_{i}\frac{h^{2}}{2m_{i}}\nabla_{i}^{2}-\sum_{iI}\frac{e^{2}Z_{I}}{\left|R_{I}{\;-\;r}_{i}\right|}+\frac{1}{2}\sum_{ij(i\neq j)}\frac{e^{2}}{\left|r_{i}{\;-\;r}_{j}\right|}+\frac{1}{2}\sum_{IJ(I\neq J)}\frac{Z_{I}Z_{J}e^{2}}{\left|R_{I}{\;-\;R}_{J}\right|}$$

where *h* corresponds to approximate Planck’s constant, *M_I_* represents the mass of the *I*-th nucleus, *m_i_* represents the mass of the *i*-th electron, *Z_I_* represents the number of electrons carried by the *I*-th nucleus, *Z_J_* represents the number of electrons carried by the *J*-th nucleus, **R***_I_* represents coordinates of the *I*-th nucleus, **R***_J_* represents coordinates of the *J*-th nucleus, *r_i_* represents coordinates of the *i*-th electron, and *r_j_* represents coordinates of the *j*-th electron.

First-principles calculations mean that the ground state energy of the system can be solved by self-consistency without using any empirical parameters in the calculation process. The process only uses the fundamental physical constants, such as the charge and mass of the electron, and it obtains the physical properties of the graphene PD sensor material in electricity.

### 2.1. Density Functional Theory

The basic idea of density functional theory (DFT) is that the physical properties of ground states of atoms, molecules and solids can be described by particle density functions. The density functional theory is mainly based on two theorems proposed by Hobenberg and Kohn [21].

The first theorem establishes that a given electron density uniquely defines the external potential. Consequently, since both the external potential and the Hamiltonian are determined, the wave function of the system is also completely specified.

The second theorem states that due to the determined external potential, the ground state energy of the system is the minimal value of the generalized energy.

The ground state energy is shown as Equation (3):

(3) $$E\left[\rho \right]=\int V(r)\rho (r)dr+T\left[\rho \right]+\frac{1}{2}\iint \frac{\rho (r)\rho (\;r\;\prime )}{\left|r-r\;\prime \right|}drd\;r\;\prime +E_{xc}\left[\rho \right]$$

where *ρ*(**r**) represents electron density function, and *V*(**r**) represents external field potential. The first term in Equation (3) represents the nucleus–electron interaction, the second term represents the kinetic energy of the electron, the third term represents the Coulomb interaction energy between the electrons, and the fourth term represents the exchange binding energy between the electrons. However, there are still some shortcomings in the Hobenberg–Kohn theorem. For example, the density distribution of electrons, the kinetic energy of electrons and the exchange-related generalization of electrons are unclear. Kohn and Sham proposed that the charge density can be expressed as a function of the electron wave function, which is obtained as Equation (4):

(4) $$\rho \left(r\right)={\sum_{i}\left|\phi_{i}\left(r\right)\right|}^{2}$$

where *φ*(**r**) represents the single electron wave function. This equation solves the problem above, and the Kohn–Sham equation can be expressed as Equation (5):

(5) $$H\left(r\right)=-\frac{h^{2}}{2m}\nabla^{2}+V_{ks}\left(r\right)$$

where *V_ks_*(**r**) represents effective potential, which can be expressed as Equation (6):

(6) $$\begin{array}{c} V_{ks}\left(r\right)=V\left(r\right)+\int d\;r\;\prime \frac{\rho \left(\;r\;\prime \right)}{\left|r-r\;\prime \right|}+\frac{\delta E_{xc}\left[\rho \right]}{\delta \rho \left(r\right)}\\ =V\left(r\right)+V_{coul}\left(r\right)+V_{xc}\left(r\right) \end{array}$$

where *V_coul_*(**r**) represents the Cullen role potential and *V_xc_*(**r**) represents the exchange-correlation potential.

### 2.2. Non-Equilibrium Green’s Function

The calculation of density functional theory based on non-equilibrium Green’s function (NEGF) mainly adopts the self-consistent field theory, which is similar to density functional theory, to deal with Hamiltonian quantities and electronic structure. The density matrix in a non-equilibrium state can be solved by the non-equilibrium Green’s function. The open transport boundary conditions and electrostatic boundary conditions can be described by real-space numerical equations.

The calculation steps are: initially inputting charge density, calculating Hamiltonian quantity in combination with model electrodes, calculating non-equilibrium Green’s function, then constructing a new charge density, and finally judging whether it has reached self-consistency. If not, recalculate the Hamiltonian quantity until it becomes self-consistent, then calculate other physical quantities again.

The current through the molecular junction is obtained by calculating the Landauer–Büttiker formula [22]:

(7) $$I=\frac{2e}{h}\int T\left(E\right)\left[f\left(E-\mu_{L}\right)-f\left(E-\mu_{R}\right)\right]dE$$

where *e* represents electron charge, *h* represents Planck’s constant, *f* represents the Fermi function, *μ_L/R_* is the electrochemical potential of the left and right electrodes, *μ_L/R_* = *E_F_* is set as the Fermi energy level.

The transport coefficient *T*(*E*) of the model is the sum of the electron deficiency transport rate and the transport probability of all possible orbits, which is shown in Equation (8) [23]:

(8) $$T\left(E\right)=T_{r}\left\lfloor \Gamma_{L}\left(E\right)G^{R}\left(E\right)\Gamma_{R}\left(E\right)G^{A}\left(E\right)\right\rfloor$$

where *T_r_* represents matrix tracing, *G^R^*^/^*^A^* is the advance and delay Green’s function of the extended molecule, and *Γ_L_*_/_*_R_* is the coupling matrix between the middle scattering region and the left and right electrodes.

The transport coefficients are important parameters in the electric transport calculation, and the electric transport capacity of the model can be determined by the transport coefficient curves.

## 3. Microscopic Model of New Graphene Partial Discharge Sensor

In order to further study the charge parameter characteristics of the graphene PD sensor, the traditional PD sensor and the new graphene PD sensor are compared and analyzed. The model is constructed for the copper electrode plate of a traditional PD sensor and the graphene copper-clad electrode plate of a graphene PD sensor.

An initial model consisting of the number of atoms that constitute the analytical model is first created. For the convenience of calculation, the small cells in the periodic structure are selected for modeling calculation since the models are all periodic structures, and so the size of the model does not affect the calculation results. The coordinates of the individual atoms of the initial structure and the cell constants (a, b, c and a, β, γ) are adjusted by structural optimization, which helps the model parameters to be closer to the actual structural parameters. Then, it can be optimized to the minimum energy as the most stable configuration. The calculations were performed using Materials Studio (version 2019, Dassault Systèmes BIOVIA, San Diego, CA, USA). The structure was optimized by using the CASTEP package with the generalized gradient approximation (GGA) under the Perdew–Burke–Ernzerhof parameterization (PBE) for the exchange of correlation functions [24]. Van der Waals interactions were incorporated via Grimme’s DFD-D scheme [25]. All calculations used a supersonic pseudopotential and a 320 eV plane wave cutoff. The Brillouin zone was sampled by 1 × 1 × 1 k points based on the Monkhorst–Pack method [26]. The maximum residual displacement of the atoms is 0.002 A. The maximum residual force is 0.05 eV/Å. The maximum force per atom is 0.1 GPa. The energy convergence criterion is 2 × 10^−5^ eV/atom. The self-consistent field calculation tolerance is 1 × 10^−5^ Ha. In this way, the geometry can be optimized to converge.

**Figure 1:**
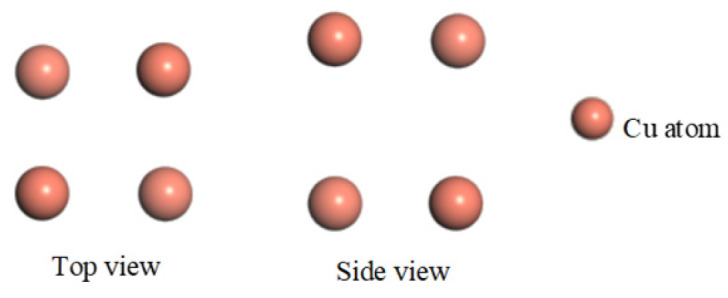
Microscopic model of traditional PD sensor electrode plate.

A microscopic model of a copper crystal was built for the copper electrode plate of a traditional PD sensor with an atomic number of 4. The optimized model is shown in Figure 1.

**Figure 2:**
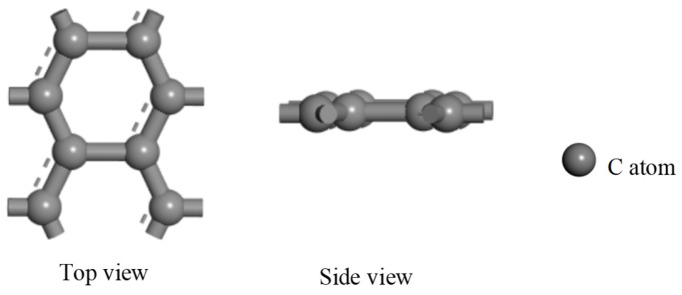
Microscopic model of graphene.

The graphene and copper constituents of the new PD sensor’s electrode must be modeled separately at the microscopic level. This bottom-up approach is then followed by modeling the composite system. The copper part is consistent with the microscopic model of copper crystals. As for the graphene model, it has an atomic number of 8 and a vacuum thickness of 20 Å to avoid mutual interlayer interactions. The optimized model is shown in Figure 2.

**Figure 3:**
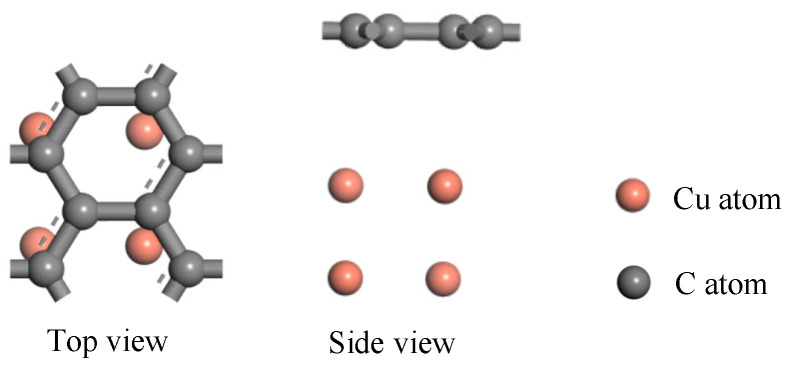
Microscopic model of graphene PD sensor electrode plate.

According to the graphene model and copper model, the microscopic model of a graphene copper-clad electrode plate with atomic number 12 is achieved with a heterojunction structure [27]. The lattice mismatch in the monolayer graphene–copper electrode system remains below the critical threshold required for interfacial stability, thereby eliminating concerns regarding interfacial strain and defect generation [28]. The optimized model is shown in Figure 3.

The optimization models are based on the energy minimum principle, which is used to determine the most stable position of the material system and the lattice constant of the system. Based on these steps, structural optimization is performed to obtain the most stable structure, which is a necessary prerequisite for the physical property analysis of PD sensor materials.

## 4. Calculation of Electrical Charge Properties

The population analysis of electrical charge properties of the two electrode plates, such as energy band, density of states, charge density and Mulliken charge, was carried out using density functional-based electrical property calculations. Both the microscopic electron activity sequence and electron transfer behavior of the two materials were also studied.

To compare their electrical charge characteristics, the optimized structures of the traditional copper electrode and the graphene-clad copper electrode were evaluated separately. In all DMOL^3^ calculations, the exchange-correlation interactions were analyzed by the GGA-PBE method. The DFD-D scheme proposed by Grimme was used to correct van der Waals forces [25]. The convergence criterion is: set SCF tolerance as 1 × 10^−4^ Ha, set MIN as basis group, set all electrons as core treatment, set monopole as multipolar expansion, and set the k-point grid to 1 × 1 × 1 [26].

A comprehensive analysis was conducted on both systems, aiming to compare their electrical charge properties through an in-depth investigation of electronic characteristics, charge distribution patterns, and microscopic electron dynamics. With the aim of simulating the electric fields induced by actual PD conditions, six sets of electric fields were applied including 0 a.u., 2 × 10^−12^ a.u., 0.001 a.u., 0.002 a.u., 0.003 a.u., 0.004 a.u. (1 a.u. = 5.142 × 10^11^ V/m) [29]. Real partial discharge simulations usually set the electric field to 1 V/m, and so the electric field value of 2 × 10^−12^ a.u. was chosen. The electrical charge properties of a traditional PD sensor electrode plate and a graphene PD sensor electrode plate were compared under the six sets of electric fields.

### 4.1. Electronic Structure

**Figure 4:**
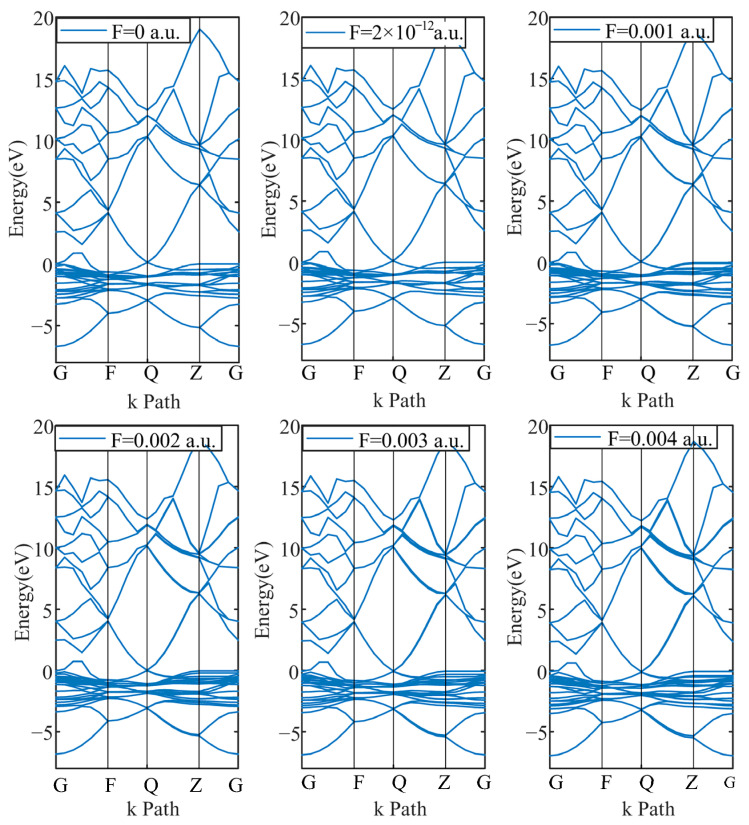
Energy bands of a traditional PD sensor electrode plate under different electric fields.

**Figure 5:**
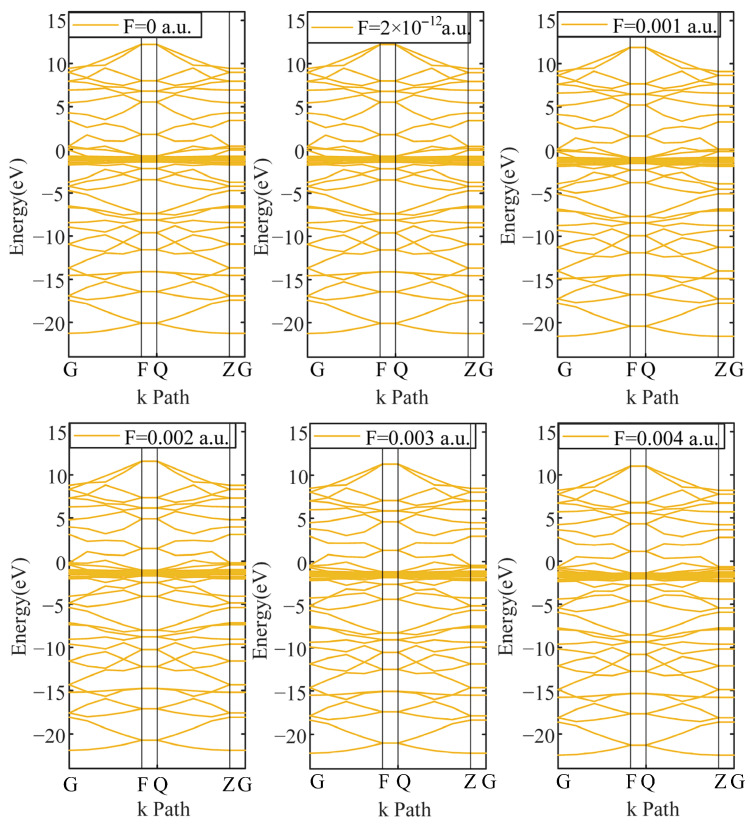
Energy bands of a graphene PD sensor electrode plate under different electric fields.

The electronic structure can be characterized by the energy band and density of states. The energy band mainly determines the electronic properties of materials. By observing the energy gap between the energy bands, the basic properties of the material can be determined, such as whether it is an insulator, a conductor or a semiconductor. The energy band structure is shown in Figure 4 and Figure 5.

Figure 4 and Figure 5 show that the energy bands of the traditional PD sensor electrode plate and graphene PD sensor electrode plate are different, but neither of them displays energy band gaps. Both electrode materials demonstrate metallic band characteristics, which renders them highly conductive. This intrinsic property enables them to serve as effective PD sensor electrodes, capable of coupling the electrical signals produced in real partial discharge environments. Furthermore, the energy band does not change with the change in electric fields.

**Figure 6:**
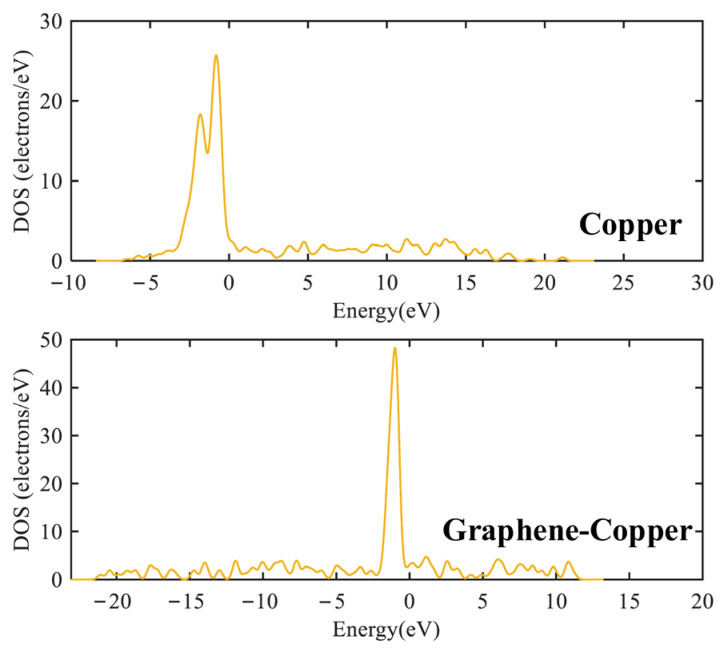
DOS for traditional and graphene PD sensor electrode plates at F = 2 × 10^−12^ a.u.

**Figure 7:**
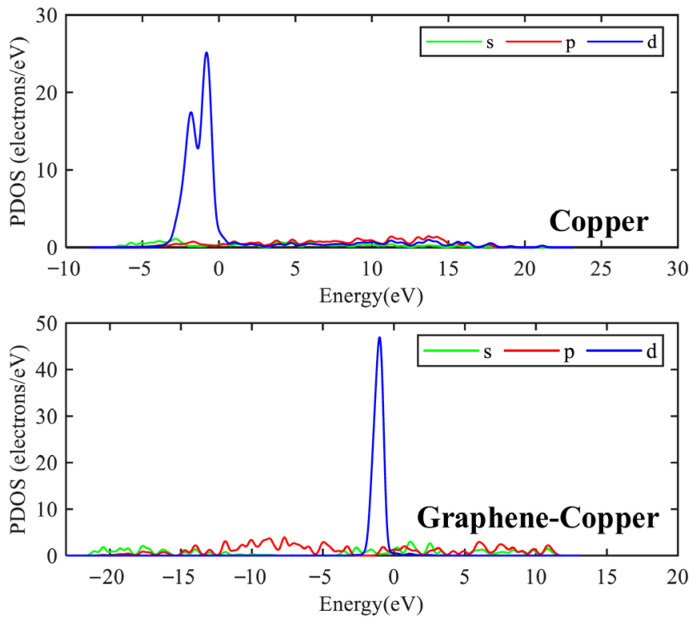
PDOS for traditional and graphene PD sensor electrode plates at F = 2 × 10^−12^ a.u.

Electronic structure analysis can be performed using the density of states (DOS) and partial density of states (PDOS). DOS counts the relative number of energy levels in each energy range, which provides a useful tool for qualitative analysis [30]. In addition, the contribution of a particular atomic orbital s, p, d or f can be determined by PDOS. The DOS and PDOS were calculated for traditional and new graphene PD sensor electrode plates at F = 2 × 10^−12^ a.u. The results are shown in Figure 6 and Figure 7.

Figure 6 shows that the traditional and graphene PD sensor electrode plates have non-zero DOS values at the Fermi energy level (energy = 0 eV), with two major peaks at −0.8 eV and −1.1 eV, respectively. The presence of finite electronic states at the Fermi level confirms that both materials exhibit metallic conductivity, which is essential for facilitating charge transfer in the sensing process. Compared with the traditional PD sensor electrode plate, the graphene PD sensor electrode plate exhibits a larger density of states near the Fermi energy level, indicating a higher carrier concentration and better metallicity, which is beneficial for promoting charge transfer.

To gain deeper insight into the origin of this electronic conductivity, a PDOS analysis was performed, in which the total DOS was decomposed into specific orbital contributions. This approach enables the identification of the electronic orbitals primarily responsible for the states near the Fermi level. As shown in Figure 7, the d orbital is found to have the largest contribution in the traditional plate, whereas for the graphene-based plate, the electronic states near the Fermi level are observed to originate predominantly from the p orbitals. Physically, this indicates that the conductive behavior in the graphene electrode is governed by the out-of-plane π-electrons. Owing to the carbon–carbon sp^2^ hybrid structure and the formation of extensive delocalized π-bonds, enhanced densities of states for both s and p orbitals are observed in the graphene-based PD sensor electrode plate. Notably, the peak near the Fermi level is more pronounced for the p orbital than for the s orbital, and the density of states associated with the p orbital is substantially higher than that of the s orbital. From the perspective of the sensing mechanism, this orbital composition is critical. The extensive overlap of p orbitals (forming π-bands) provides a high-mobility pathway for charge carriers. When graphene interacts with the traditional electrode plate surface, the delocalized π-electron network facilitates rapid electron transfer under an electric field, potentially enhancing the sensor’s response speed and sensitivity. The comparative analysis confirms that the graphene-based electrode exhibits superior electrical conductivity, primarily due to the active participation of its p-electronic states.

### 4.2. Charge Density

**Figure 8:**
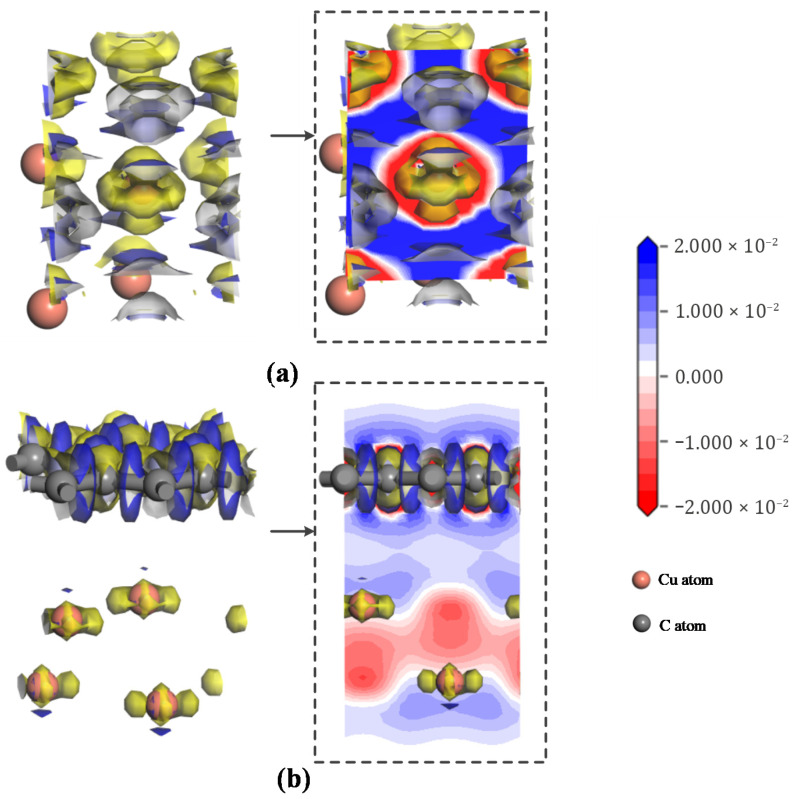
Differential charge density of traditional and graphene PD sensor electrode plates at F = 2 × 10^−12^ a.u. (**a**) Graphene–copper; (**b**) Copper.

In terms of charge density, the charge difference density can visualize the charge transfer. And the electron charge density difference is also used to illustrate the gain and loss of electrons during the interaction. The differential charge densities of traditional and graphene PD sensor electrode plates are shown in Figure 8.

Figure 8a depicts the differential charge density of the conventional PD sensor electrode plate, and Figure 8b presents that of the graphene-based PD sensor electrode plate. In Figure 8, the left panel depicts electron density distribution, with yellow and blue regions corresponding to electron deficiency and enrichment, respectively. The right panel provides a cross-sectional view, where red and blue similarly indicate deficiency and enrichment. The lighter color means that charge transfer occurs in the electrode plates, showing greater interaction energy and charge transfer. Figure 8a shows that the graphene PD sensor electrode plate is in a higher state of accepting or providing electrons than the traditional PD sensor electrode plate, i.e., Graphene–copper > Copper. It means that once graphene is added to form a graphene PD sensor electrode plate, charge transfer occurs between the two materials, with electron enrichment in the graphene side and electron deficiency in the copper side. Electrons are also transferred from the copper electrode plate to the graphene. The analysis indicates that the graphene-based PD sensor electrode exhibits significantly higher interaction energy and greater charge transfer under the simulated partial discharge electric field.

### 4.3. Charge Distribution

The population analysis can be used to analyze the charge distribution of materials. It characterizes the electron distribution within individual atomic orbitals, enabling the quantitative analysis of charge transfer. Additionally, the Mulliken charge results can be used to understand the entire charge redistribution of the system. The electron charges obtained by each Cu atom in the electric field of F = 0 a.u., 0.001 a.u., 0.002 a.u., 0.003 a.u. and 0.004 a.u. are shown in Table 1.

Table 1 shows that Cu atoms do not carry charges when the electric field is 0, while the graphene PD sensor electrode plate carries more charges. The underlying mechanism involves charge transfer at the graphene–metal interface upon its introduction. As for the effects of different electric fields, the charge gradually decreases when the electric field increases because the external electric field changes the charge distribution in the model space. Specifically, under a higher electric field, the charge carried by Cu atoms will decrease, which means the charge of electrons in graphene increases. Electron transfer occurs from copper to graphene, as confirmed by the corresponding charge density difference analysis. The charge transfer of Graphene–copper sensing material is at the largest (0.052 e) when the electric field is 0 a.u., indicating that the charge transfer ability is the strongest when in a lower electric field.

## 5. Transport Properties Based on the First Principles

Through the first-principles study of the PD sensor’s transport characteristics, the electric transport capacity of the graphene-based electrode can be described by calculating its transport coefficient curve. As an important tool to characterize the transport characteristics, the transport coefficient can predict the transport properties of the PD sensor. Subsequently, the electric transport capabilities of the two distinct electrode plates are assessed and compared under the influence of an actual PD electric field. In this way, it is possible to determine the transport characteristics of PD sensors from the charge level.

### 5.1. Electrical Transport Modeling

**Figure 9:**
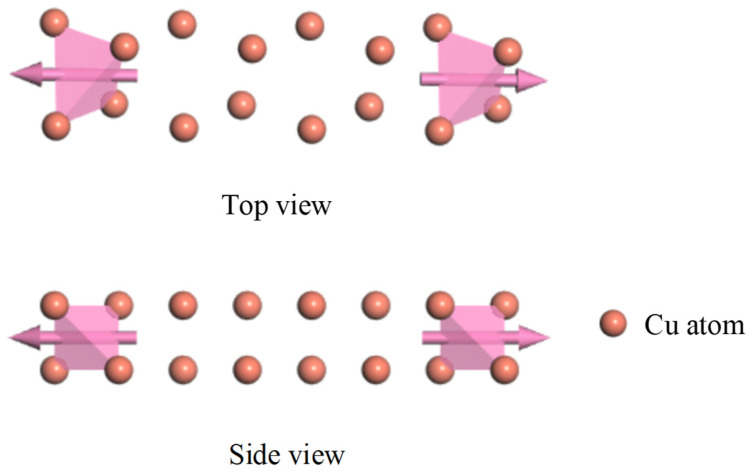
Transport model of traditional PD sensor electrode plate.

**Figure 10:**
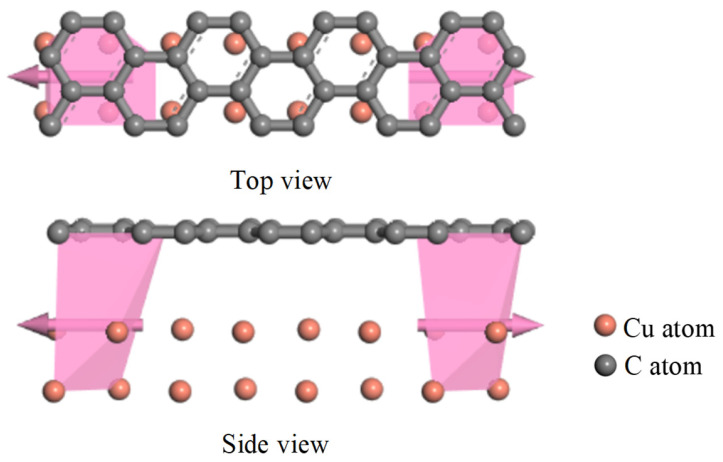
Transport model of graphene PD sensor electrode plate.

The electric transport calculation requires further supercells for the optimization of the transport model. The transport model includes three parts, which are the left electrode, middle scattering area and right electrode. The transport direction is the x-direction. The calculation is executed in two primary stages. First, the electrodes are solved self-consistently to obtain an effective potential, which is constructed by summing over all occupied states following the Fermi distribution. Second, with this potential serving as the boundary condition, a self-consistent solution is sought for the density matrix and scattering wave functions in the central scattering region. The comparison of electrical transport properties proceeds once the prescribed convergence criterion is met, at which point the requisite transport coefficient is obtained, enabling a quantitative evaluation. The transport model is shown in Figure 9 and Figure 10.

Figure 9 depicts the charge transport model of the conventional PD sensor electrode plate, and Figure 10 presents that of the graphene-based PD sensor electrode plate. In this research, the electric transport calculations were carried out using the software package DMOL^3^ in Material studio 2019, which is based on the combination of DFT and NEGF. The exchange-correlation interactions were described using the Perdew–Burke–Ernzerhof (PBE) formulation of the generalized gradient approximation (GGA). The MIN basis group was chosen for all atoms. For the dispersion corrections, Grimme’s DFT-D scheme was applied [25]. For the core electron treatment, the DSPP pseudopotential was employed. The Monkhorst–Pack scheme with a 5 × 1 × 1 k-point grid was used for the special point sampling in the Brillouin zone [31]. The convergence criteria were as follows: Mixing amplitude as 0.02, multipolar expansion as Monopole, SCF tolerance as 1 × 10^−4^ Ha, the energy range as [−1 eV, 1 eV].

### 5.2. Electrical Transport Analysis

**Figure 11:**
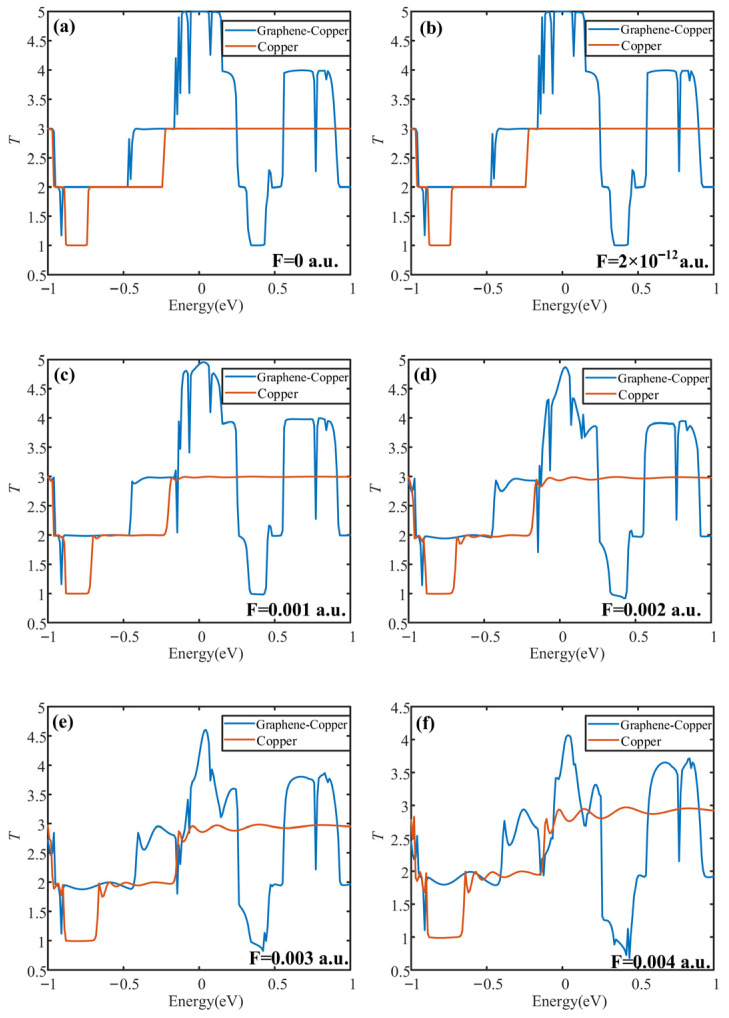
Transport curves of PD sensor electrode plate under different electric fields. (**a**) F = 0 a.u.; (**b**) F = 2 × 10^−12^ a.u.; (**c**) F = 0.001 a.u.; (**d**) F = 0.002 a.u.; (**e**) F = 0.003 a.u.; (**f**) F = 0.004 a.u.

As an important tool for characterizing the transport properties, the transport coefficient is able to predict the electron transport properties of the model, which can directly affect the detection ability of the PD sensor. Consequently, separate electrical transport models were constructed for the traditional and the graphene-clad PD sensor electrode plates. To this end, a series of six electric fields were applied to simulate the conditions arising from an actual partial discharge environment, including F = 0 a.u., 2 × 10^−12^ a.u., 0.001 a.u., 0.002 a.u., 0.003 a.u., 0.004 a.u. The transport coefficient curves of both traditional and new graphene PD sensor electrode plates under different electric fields were calculated, and are shown in Figure 11.

Figure 11 also shows that there is no transport peak near the Fermi energy level (0 eV) in the traditional PD sensor electrode plate. While there is a clearly visible transport peak in the graphene PD sensor electrode plate with a value of 5, it is about 66% higher than the electrode plate of the traditional PD sensor. Owing to its higher carrier mobility, the graphene PD sensor electrode exhibits a larger effective integral area under each applied electric field compared to the traditional electrode. This enhanced transport characteristic directly indicates its superior electric transport capacity. When applied to partial discharge detection, the transport characteristics of graphene PD sensors are stronger for electrical signals in partial discharge electric fields.

## 6. Experimental Verification

Experiments were carried out to verify the charge parameter properties of the graphene PD sensor analysis method. The transient earth voltage sensor was chosen primarily because of its structural simplicity and ease of modification. By altering only the electrode plate material—while all other structural parameters and the underlying sensing mechanism were kept unchanged—a controlled variable approach was employed to isolate and investigate the effect of the electrode material. This experimental design allowed the graphene-based sensor to be directly compared with the conventional copper-based sensor, thereby providing an initial experimental validation of the atomic-scale charge transfer characteristics predicted by the first-principles calculations and simulations. In this experiment, two TEV partial discharge sensors of model HOPD-9209B with identical structural parameters were chosen. The main technical parameters of the transient earth voltage partial discharge sensor are shown in Table 2.

**Figure 12:**
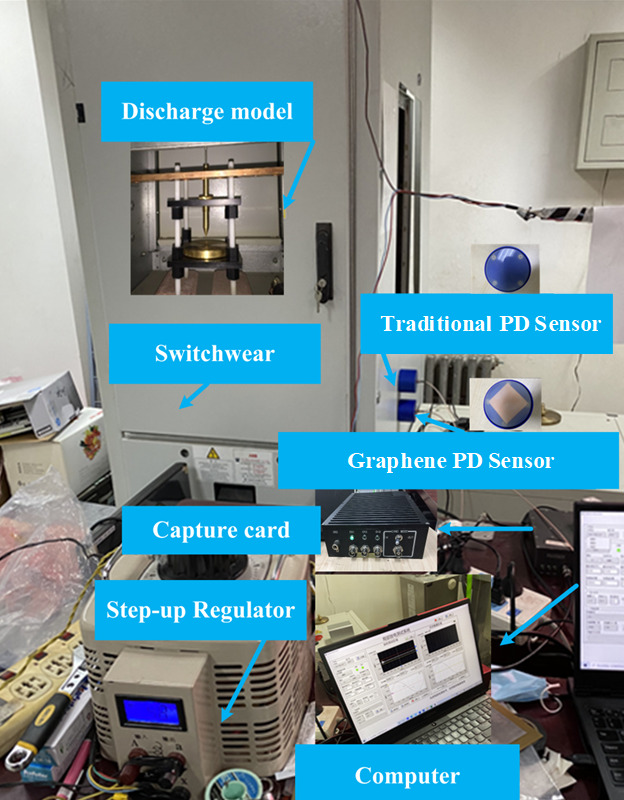
Partial discharges detection platform.

**Figure 13:**
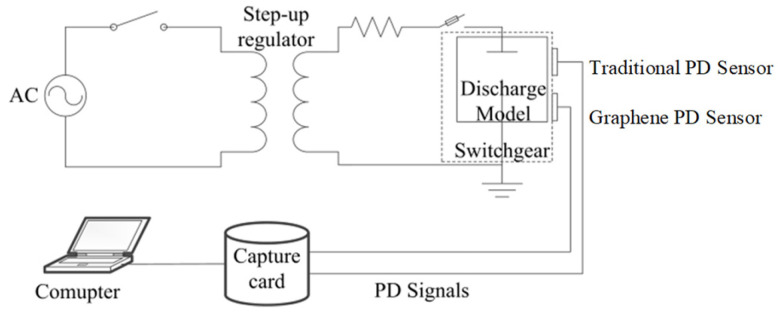
Schematic diagram of the detection platform.

A partial discharge signal detection platform was built in the laboratory. The primary components of the detection platform are an AC power supply, a step-up regulator, a partial discharge excitation model within a switchgear, two sensors, a data collector, a data acquisition card, and a computer. A traditional PD sensor (sensor 1: electrode plate material is copper) and new graphene PD sensor (sensor 2: electrode plate material is graphene copper-clad) were selected. The discharge model refers to the simulation of partial discharge defects, which can be used to generate discharge signals. The Corona discharge model was used in the platform because it is common in power systems. The main equipment and hardware connection of the experimental platform are shown in Figure 12 and Figure 13.

Figure 12 shows the partial discharge detection platform. The 220 V AC power supply serves as the input for the voltage regulator. The step-up regulator with a transformation ratio of 1:110 is used to boost voltage and excite the partial discharge model in the switchgear. Two partial discharge sensors (Sensor 1: traditional copper plate; Sensor 2: graphene-coated copper electrode plate) were used to detect partial discharge signals. A data collector, data acquisition card, and computer were used for data analysis.

**Figure 14:**
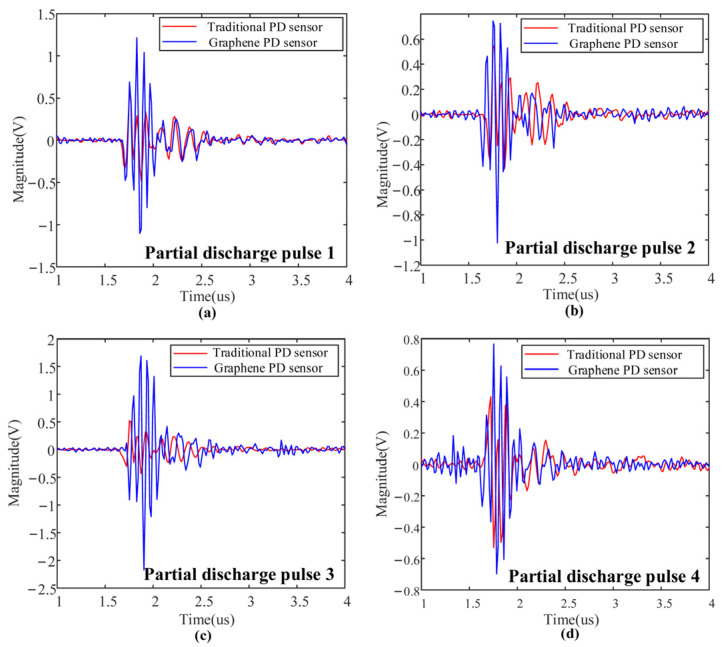
Partial discharge waveform detected by different sensors. (**a**) Partial discharge pulse 1; (**b**) partial discharge pulse 2; (**c**) partial discharge pulse 3; (**d**) partial discharge pulse 4.

At a regulated voltage of 48 kV, partial discharges are generated within the switchgear model. These resulting signals are subsequently detected by the sensors integrated with the two distinct electrode plates. The actual electrical transport ability can be determined by analyzing the partial discharge signals. For four sets of partial discharge pulses, the discharge waveform detected by PD sensor 1 and PD sensor 2 are shown in Figure 14.

Figure 14 demonstrates that across four experimental sets, PD Sensor 2 (Graphene–copper) consistently exhibits a significantly higher response amplitude to the identical PD pulse compared to Sensor 1 (Copper). Quantitatively, the signal amplitude of Sensor 2 is approximately 40% higher than that of Sensor 1 across all test sets. This performance enhancement is primarily attributed to the superior charge transport capability conferred by the graphene coating. Specifically, the high electron mobility and unique carrier transport properties of graphene effectively facilitate the rapid collection and transmission of charge generated by PD events. This empirical finding is in strong agreement with the conclusions drawn from our first-principles analysis regarding the improved electrical transport properties of the graphene-clad electrode, thereby validating the theoretical prediction at the device level.

## 7. Conclusions

For the improvement of sensing materials in partial discharge (PD) detection, this research proposes a charge parameter analysis method for graphene-based PD sensors grounded in first-principles calculations, with complementary experimental characterization of device-level response. Both systematic simulation results and experimental validation revealed the better transfer properties of the graphene PD sensor. The principal conclusions of this study are summarized as follows:

(1) Compared to conventional copper-based electrode materials, the graphene–copper composite electrode exhibits superior charge-related characteristics, with the graphene sensor achieving a charge transfer of approximately 0.052 e.
(2) Regarding electrical transport properties, the graphene PD sensor demonstrates a transport coefficient approximately 66% higher than that of its traditional counterpart.
(3) Experimental results further reveal that the PD sensor incorporating a graphene-clad copper electrode achieves markedly improved detection sensitivity—approximately 40% higher than conventional sensors—along with enhanced electrical transport capability for PD signals.

Collectively, the proposed graphene PD sensor demonstrates significant advantages in charge capture capability, electrical transport efficiency, and detection sensitivity compared to conventional copper-based PD sensors. These improvements provide a reliable technical solution for high-precision insulation monitoring in power grids.

This study focuses on charge-level characterization, verification, and theoretical framework, but has limitations. A full quantitative model linking microscopic charge properties to macroscopic performance still requires further investigation. The key points for future research are as follows: (1) Construct an equivalent-circuit model of the graphene-based PD sensor that integrates DFT-calculated charge parameters to establish a quantitative bridge between microscopic charge properties and macroscopic circuit performance; (2) Conduct systematic circuit simulations and experimental tests to analyze the synergistic effects of charge-level characteristics, electromagnetic coupling, and load parameters on sensor sensitivity.

## Figures and Tables

**Table 1:** Mulliken charge population analysis of traditional and graphene PD sensor electrode plate under different electric fields (unit: e).

Electric Fields (a.u.)	Material	Cu Atom1	Cu Atom2	Cu Atom3	Cu Atom4	Total
F = 0	Copper	0	0	0	0	0
	Graphene–copper	0.019	0.021	0.006	0.006	0.052
F = 0.001	Copper	0.011	0.011	−0.011	−0.011	0
	Graphene–copper	0.018	0.019	0.003	0.003	0.043
F = 0.002	Copper	0.022	0.022	−0.022	−0.022	0
	Graphene–copper	0.016	0.017	0.001	0.001	0.035
F = 0.003	Copper	0.033	0.033	−0.033	−0.033	0
	Graphene–copper	0.015	0.016	−0.001	−0.001	0.029
F = 0.004	Copper	0.044	0.044	−0.044	−0.044	0
	Graphene–copper	0.015	0.015	−0.002	−0.002	0.026

**Table 2:** The main technical parameters of the transient earth voltage partial discharge sensor.

Main Technical Parameters	Value
Detect bandwidth	3 M~100 MHz
Measurement range	0~60 dB
Measurement error	±1 dB
Maximum pulses per cycle	720
Output interface	Output interface standard SMA

## Data Availability

The original contributions presented in this study are included in the article. Further inquiries can be directed to the corresponding author(s).
